# Editorial: New Microenvironments for Neuronal Differentiation

**DOI:** 10.3389/fnins.2022.902682

**Published:** 2022-04-26

**Authors:** Marzia Lecchi, Fabrizio Gelain

**Affiliations:** ^1^Department of Biotechnology and Biosciences, University of Milano-Bicocca, Milan, Italy; ^2^Center for Nanomedicine and Tissue Engineering, ASST GOM Niguarda and Tissue Engineering Unit, ISBREMIT, IRCCS Casa Sollievo della Sofferenza, Niguarda Ca' Granda Hospital, Milan, Italy

**Keywords:** neuronal differentiation, microenvironment, cell matrix interaction, biocompatible polymers, nervous tissue regeneration

The advancement of regenerative medicine for the treatment of spinal cord injuries and neurodegenerative diseases requires cellular models able to reproduce the complexity of cell-cell and cell-matrix interactions that occur *in vivo*. In these models, stem cells represent the most suitable cellular type because they are capable of both self-renewal and differentiation into specialized cells. However, their behavior is strictly influenced by the environment they are included in, and biocompatible polymers, scaffolds, and hydrogels have shown the capability to favor their survival and maturation.

Well-defined hydrogels, polymeric networks of synthetic or natural origin, able to swell in water, offer several advantages to stem cell maintenance, and have been particularly exploited in the last years because they can be modified in their composition to gain biochemical and mechanical properties that mimic the natural extracellular environment. Natural polymers, used to derive hydrogels, are collagen, hyaluronic acid, chitosan or gelatin. Functionally active peptides, derived from extracellular matrix (ECM) molecules, such as fibronectin, vitronectin, and collagens are also employed in hydrogel constitution and are known to promote neurite outgrowth.

Synthetic hydrogels have emerged as highly tunable biomaterials which minimize unintentional cell responses and offer pathogen-free regenerative approaches. For this reason, they are good candidates for implantable grafts (Unal and West, 2020). Here Glotzbach et al. provide an overview on the application of synthetic hydrogels, based on polyacrylamides and modified with cationic moieties and/or with the fibronectin derived RGD sequence, for supporting neural stem cell (NSC) development and maintenance. Polycationic polymers are known to influence NCS behavior. Moreover, the RGD (Arg-Gly-Asp) peptide and other functional motifs, discovered in other ECM molecules, are known to sustain cell attachment and to promote neurite outgrowth: they represent promising tools in regenerative medicine since they can more specifically trigger NSC response. Synthetic hydrogels, with cationic moieties and functionalized with the RGD peptide, can reproduce the stiffness of brain tissue, and take advantage of the synergic effects of the cationic and RGD motifs, that take place on different distances and time scales.

The evolution of 3D hydrogels, which mimic more accurately the biochemical and mechanical properties of cell environment, has highlighted the advantages of hydrogels based on self-assembling peptides (SAPs). Marchini et al. previously developed a self-assembling peptide scaffold which was able to guide the maturation of human neural stem cells (hNSCs) into mature neurons showing different phenotypes and neurotransmitters (Marchini et al., 2019). Here they demonstrate that their multifunctionalized hydrogel (HYDROSAP) could induce serum-free long-term 3D cultures of distinct hNSC lines to progressively differentiate and maturate, generating networks with increasing expression of GABAergic, glutamatergic, and cholinergic neuronal phenotypes. In these 3D models oligodendrocytes also developed and were able to form insulating myelin sheaths, demonstrating the possibility to successfully standardize a 3D neural culture to study neuronal differentiation and the progressive maturation.

Moreover, Fannon et al. demonstrate that scaffolds of alginate hydrogels of different molecular weights could guide mouse ESCs (embryonic stem cells) to differentiate into embryonic body-like-aggregates, including cells of the three germ layers.

Finally, brain on chip platforms represent a new step toward neuronal cell models to understand brain in both physiological and pathological conditions. These systems allow to establish compartmentalized regions in which it is possible to replicate the nano-architecture of proteins in the extracellular matrix. Microfluidic compartmentalization and nanotopography (nanogrooves and microtunnels) by Bastiaens et al. provided a geometric confinement of the neurites separated from the bodies and could influence cellular behavior, respectively. On this platform the human neuroblastoma SHSY5Y cell line could acquire a differentiated phenotype and could be maintained up to at least 21 days *in vitro*.

In conclusion, different systems mimicking the extracellular environment are now available that can be adopted to induce neuronal differentiation, a progressive maturation over time and the maintenance of the culture for longer period. These systems are built up according to the information which has been acquired on the extracellular matrix properties and on the complex cell-matrix interactions, and they are fundamental tools for deepening the comprehension of the environmental influence on cell behavior in both physiological and pathological conditions.

It is not a far reach goal to imagine that in the future even more refined approaches will emerge in order to provide implantable pre-cultured neural patches to tackle the regeneration of nervous tissues as well.

## Author Contributions

All authors listed have made a substantial, direct, and intellectual contribution to the work and approved it for publication.

## Conflict of Interest

The authors declare that the research was conducted in the absence of any commercial or financial relationships that could be construed as a potential conflict of interest.

## Publisher's Note

All claims expressed in this article are solely those of the authors and do not necessarily represent those of their affiliated organizations, or those of the publisher, the editors and the reviewers. Any product that may be evaluated in this article, or claim that may be made by its manufacturer, is not guaranteed or endorsed by the publisher.
